# The Role of Social Support in the Experience of Informal Caregivers of Cancer Patients: An Umbrella Review

**DOI:** 10.1002/pon.70430

**Published:** 2026-03-19

**Authors:** Sabrina Cipolletta, Martina Orsolini, Andrea Ghiani

**Affiliations:** ^1^ Department of General Psychology University of Padua Padua Italy; ^2^ IRCCS San Camillo Hospital Venice Italy

**Keywords:** cancer, caregiver, dyad, mental health, psycho‐oncology, social support, well‐being

## Abstract

**Objective:**

To understand the role of social support in the caregiving experience of cancer patients through a narrative synthesis of systematic reviews and meta‐analyses, providing a comprehensive overview in the field.

**Methods:**

A systematic search of systematic reviews and meta‐analyses published between 2013 and 2024 was conducted in PubMed, EMBASE, PsycINFO, CINAHL, Web of Science, and Scopus using PCC‐based search terms related to informal caregivers, social support, and cancer.

**Results:**

The search resulted in a total of 1377 articles, of which 32 met the inclusion criteria and quality assessment. Through a high‐level narrative synthesis, we identified three main thematic areas: (i) the role of social support in shaping caregivers' outcomes, including their psychological well‐being, adaptation to the caregiving role and difficulties in receiving and seeking help; (ii) changes in interpersonal relationships, including the relationship with the patient; and (iii) support needs and psychosocial interventions designed to address them.

**Conclusions:**

Our narrative synthesis confirms social support as a critical factor shaping psychological well‐being, role adaptation, and quality of life among informal caregivers. Evidence consistently shows that emotional and instrumental support reduce caregiver burden and distress, despite frequently reported barriers to seeking and receiving help. Caregiving reshapes interpersonal relationships, requiring ongoing role renegotiation within the caregiver‐patient dyad. Dyadic interventions focusing on relational communication are more effective than information‐only approaches and adaptable across settings. Future research and policies should prioritize person‐centered support throughout the cancer trajectory.

## 1 Background

The global increase in cancer diagnoses presents significant challenges not only for patients but also for informal caregivers who provide support [1, 2, 3, 4]. An increasing number of family members, relatives, partners or friends take on the role of informal caregivers for people affected by cancer [1]. Caregivers, primarily partners or close relatives, manage daily activities and assume multiple responsibilities in patient care, which constitutes a significant burden in their life [5]. Estimates predict a 21% increase in informal caregiver burden in Europe by 2040 and a 31% rise in annual cancer deaths [3], making it a priority for healthcare systems to adopt informed, evidence‐based policies and practices targeting the needs of this growing population.

At present, studying the experience of informal caregivers, regardless of the patient's condition, involves engaging with a substantial body of literature focused on caregivers' quality of life, which overall highlights the negative impact of cancer on informal caregiver's health [4, 6]. For example, informal caregivers frequently report sleep disturbances, fatigue, pain, weight gain and a higher vulnerability to developing depression compared to non‐caregiving individuals [7, 8, 9]. Caregiver burden varies widely and requires ongoing adjustment to changes in the patient's condition. For instance, the highly variable cancer trajectories require substantial technical and psychosocial demands on caregivers. In some cases, caregivers also live with the patient, which can further intensify these demands by increasing continuous responsibility and reducing opportunities for rest or personal time. This leads to caregiver burden being dependent on patients' physical and mental health characteristics [10]. Interestingly, research also shows that some positive aspects can be reported while caregiving, such as gratification, satisfaction, hope, personal growth, or a strengthened sense of closeness to the patient and other family members [11].

A critical factor in the overall experience of informal oncology caregivers is social support. Social support is often understood as an interactive process of giving and receiving help, based on reciprocity and on the affective dimension of a relationship. Social support is usually conceptualized as a multidimensional construct comprising four components: emotional, instrumental, informational, and appraisal support [12]. Emotional support involves empathy, acceptance, and reassurance, while instrumental support includes tangible assistance such as time, money, or practical help, an aspect shown to be crucial in reducing oncology caregiver burden [1, 13]. Informational support, which provides guidance for managing stressful situations, is particularly important during the initial phase of cancer [14]. Appraisal support consists in offering feedback promoting self‐evaluation and reinforcing personal identity. Together, these forms of support seem to play a critical role, influencing caregiver burden, stress, health outcomes, and psychological well‐being, including anxiety and depression [14]. Conversely, insufficient social support is recognized as a risk factor that may also hinder effective grief processing [15, 16]. Social support also differently impacts the caregiver experience based on gender differences. Overall, male caregivers more often report a positive oncology caregiving experience and lower stress, regardless of perceived competence or external support [17], whereas female caregivers (especially adult daughters) tend to experience higher stress and poorer quality of life [17, 18]. Across the majority of studies, male caregivers are primarily spouses or partners, while female caregivers more frequently cover a wider range of caregiving relationships beyond spousal ones (e.g., daughters) [19]. Female caregivers seem to experience a decrease and impoverishment of their social network compared to men [18]. This difference has been linked to male caregivers being more prone to increase their network by delegating tasks, often involving female relatives, whereas women are less inclined to ask for or delegate instrumental support [20].

Overall, the scientific literature provides a substantial number of systematic reviews and meta‐analyses examining the experiences of informal cancer caregivers, particularly the role of social support and its influence on caregiver outcomes such as burden, anxiety and depression, changes in personal identity, and adaptation to the caregiving role. Given the relevance of this topic for informed policy and practice, this umbrella review aims to provide a narrative high‐level synthesis of the existing evidence by integrating findings from systematic reviews and meta‐analyses focused on social support among informal oncology caregivers. In line with the general aim of an umbrella review [21], the goal of our narrative synthesis is twofold: first, to provide a comprehensive overview of the field that can help researchers orient themselves within this extensive body of literature, by integrating studies examining diverse aspects of social support in the oncology caregiving experience; and second, to possibly inform policy and practice by identifying areas of consensus and highlighting possible inconsistencies. Following the PCC (*Population*, *Concept*, *Context*) framework, our study question is: what evidence from systematic reviews and meta‐analyses describes the role of social support (*Concept*) in shaping the experience of caregivers of cancer patients (*Population*) within the cancer care setting (*Context*)?

## 2 Methods

The present umbrella review collects systematic reviews and meta‐analysis, according to the Preferred Reporting Items for Systematic Reviews and Meta‐Analysis (PRISMA) [22].

### 2.1 Search Strategy

To identify all relevant publications, we conducted a systematic search in the following bibliographic databases: PubMed, EMBASE, PsycINFO, CINAHL, Web of Science, and Scopus. For each database, the search was conducted using specific keywords (Box 1) and Mesh terms (where a controlled vocabulary was available) and then combined across databases to remove duplicates. The keywords were grouped according to the elements of our PCC‐framed question: caregiver (*Population*); social support (*Concept*); cancer (*Context*). The results obtained for each topic were then combined using the Boolean operator “AND”. Our search was limited to systematic reviews and meta‐analyses (the keywords *systematic review* OR *meta‐analysis* were used). When possible, *systematic review* and/or *meta‐analysis* filters were applied. All included studies focused on adults populations aged 18 years or older.

**BOX 1 Search terms.**

1

**Table:** 

Element	Keywords
Population	caregiver* OR informal caregiver OR informal caregiving OR carer* OR family caregiver* OR home help OR home care OR friend* OR family member* OR couple* OR spouse* OR marital OR sibling* OR son OR daughter OR relative OR kin OR parent OR caregiver burden or caregiver burnout OR family relation OR sibling relation OR home care OR cancer family OR domestic partner OR husband OR wife OR sibling OR firstdegree relative OR nuclear family OR brother OR sister OR relative
Concept	social support OR psychosocial support OR social network OR social integration OR social embeddedness OR social relationship OR interpersonal exchanges OR social companionship OR support OR loneliness OR social isolation OR emotional support OR affective support OR esteem support OR intimate interaction OR marital status OR instrumental support OR tangible support OR enacted support OR informative support OR guidance OR advice OR cognitive support OR appraisal support OR appraisal support OR affirmational support OR esteem support OR received support OR perceived support OR perceived social support OR psychological distance OR psychosocial support systems OR psychosocial intervention
Context	Cancer* OR neoplasm* OR tumor* OR oncology OR psycho‐oncology

### 2.2 Eligibility Criteria

Studies were considered eligible if they met the following inclusion criteria: (i) systematic reviews or meta‐analyses focusing on the role of social support in the experience of informal oncology caregivers; (ii) both informal caregivers and patients had to belong to the adult population; (iii) studies published in English; (iv) studies published between 2013 and 2024.

### 2.3 Study Identification, Selection Process and Primary Studies Overlap

Search results were imported into Zotero software, where duplicates were removed. Two authors independently screened all potentially relevant titles and abstracts. After this first selection, the full text was screened for eligibility. Disagreement was resolved through consensus with possible assistance of an independent reviewer. The quality assessment was conducted independently by two authors using the JBI Critical Appraisal Checklist for Systematic Reviews and Research Syntheses. This tool consists of 11 questions, with four possible answers: “Yes” (Y) “No” (N) “Unclear” (NC) or “Not Applicable” (NA). We adopted a conservative inclusion criterion by considering studies sufficiently valid if the number of “Yes” responses was greater than or equal to nine out of 11 items. In cases of unclear reporting, we did not contact the authors for clarification but based our decisions solely on the information provided in the articles.

We also assessed the overlap of primary studies across the included systematic reviews and meta‐analyses using the Corrected Covered Area (CCA), which estimates the proportion of shared primary studies across reviews. We used the Graphical Representation of Overlap for OVErviews (GROOVE) tool to visually map overlap patterns across the included studies [23].

## 3 Results

### 3.1 Study Selection and Quality Assessment

**FIGURE 1:**
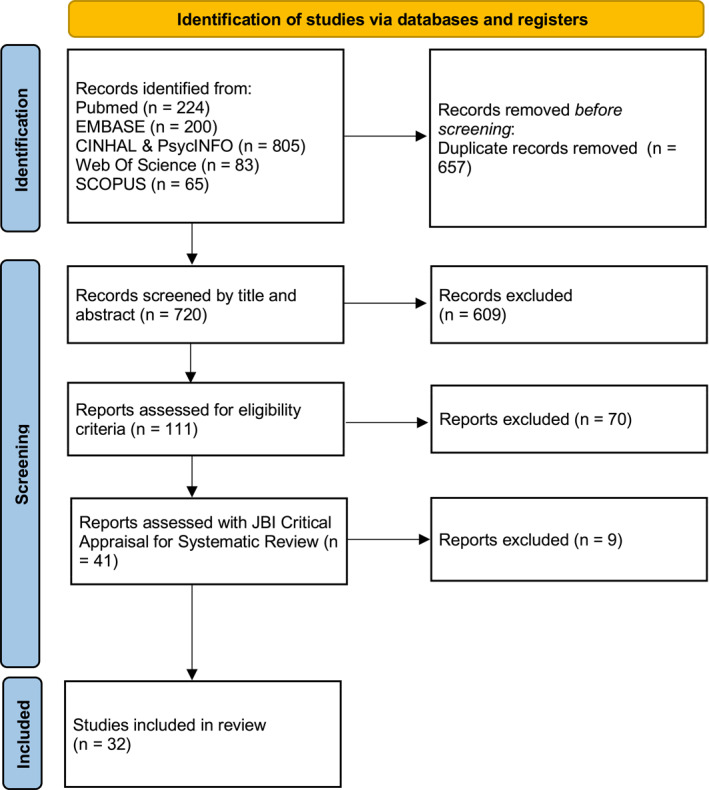
Flow chart of the search and selection process based on the PRISMA 2020 statement.

**FIGURE 2:**
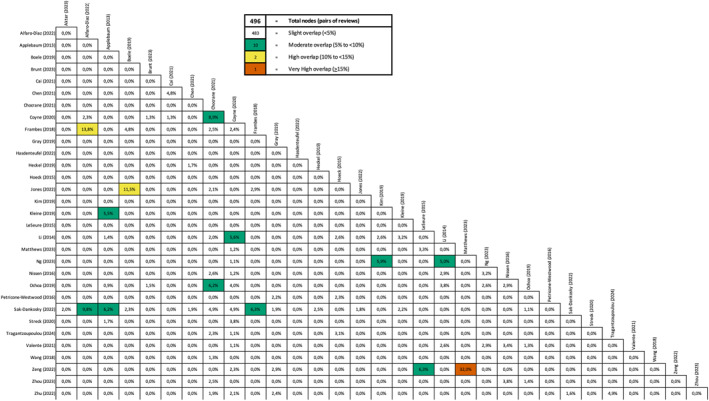
Graphical representation of overlap for overviews (GROOVE) plot illustrating the overlap of primary studies across the included systematic reviews and meta‐analyses. Overlap was quantified using the corrected covered area (CCA).

Our search strategy (Section 2.1) led to a total of 1377 studies. After removing 657 duplicates, studies were initially screened based on their title and abstract. After this first screening, 111 studies were selected for full‐text evaluation, 41 of which met all eligibility criteria (Figure 1). After quality assessment, 32 systematic reviews and meta‐analyses were considered suitable for inclusion (Table 1). The characteristics of the included studies are summarized in Table 2. The Corrected Covered Area (CCA) indicates an overall slight level of overlap across reviews (CCA = 0.58%), according to Pieper and colleagues [23]. The GROOVE plot shows no clear clusters of shared primary studies, with 483 review pairs demonstrating slight overlap, 10 pairs moderate overlap, 2 pairs high overlap, and only one pair exhibiting the highest degree of overlap (Figure 2), indicating an overall low level of redundancy among the included reviews and a limited risk of bias due to study duplication [22].

**TABLE 1:** Quality assessment of the systematic reviews and meta‐analyses using the JBI critical appraisal checklist for systematic reviews and research syntheses*. Each question (from 1 to 11) can have four possible answers: yes (Y, in green), no (N, in red), unclear (NC, in orange) or not applicable (NA, in white).

Study	JBI critical appraisal checklist* [24]											Overall appraisal
	1	2	3	4	5	6	7	8	9	10	11	
Akter et al. [25]	Y	Y	Y	Y	Y	Y	Y	Y	N	Y	Y	Include
Alfaro‐Diaz et al. [26]	Y	Y	Y	Y	Y	Y	Y	Y	N	Y	Y	Include
Applebaum et al. [27]	Y	Y	Y	Y	Y	Y	Y	Y	N	Y	Y	Include
Boele et al. [28]	Y	Y	Y	Y	Y	Y	Y	Y	Y	Y	Y	Include
1	Y	Y	Y	Y	Y	Y	NC	NC	N	Y	Y	Exclude
Brunt et al. [29]	Y	Y	Y	Y	Y	Y	Y	Y	Y	Y	Y	Include
Cai et al. [30]	Y	Y	Y	Y	Y	Y	Y	Y	Y	Y	Y	Include
Chen et al. [31]	Y	Y	Y	Y	Y	Y	Y	Y	N	Y	Y	Include
Cochrane et al. [2]	Y	Y	Y	Y	Y	Y	Y	Y	N	Y	Y	Include
Coyne et al. [32]	Y	Y	Y	Y	Y	Y	Y	Y	NC	Y	Y	Include
Frambes et al. [33]	Y	Y	Y	Y	Y	Y	Y	Y	N	Y	Y	Include
Gray et al. [34]	Y	Y	Y	Y	Y	Y	Y	Y	N	Y	Y	Include
2	Y	Y	Y	Y	Y	Y	NC	NA	NC	Y	Y	Exclude
Hasdenteufel et al. [35]	Y	Y	Y	Y	Y	Y	Y	Y	Y	Y	Y	Include
Heckel et al. [36]	Y	Y	Y	Y	Y	Y	Y	Y	N	Y	Y	Include
3	Y	Y	Y	Y	Y	NC	NC	Y	NC	Y	Y	Exclude
Hoeck et al. [37]	Y	Y	Y	Y	Y	Y	Y	Y	N	Y	Y	Include
Jones et al. [38]	Y	Y	Y	Y	Y	Y	Y	Y	N	Y	Y	Include
4	Y	Y	Y	Y	Y	NC	NA	Y	N	Y	Y	Exclude
Kim et al. [39]	Y	Y	Y	Y	Y	Y	Y	Y	Y	Y	Y	Include
Kleine et al. [40]	Y	Y	Y	Y	Y	Y	Y	Y	N	Y	Y	Include
LeSeure et al. [41]	Y	Y	Y	Y	Y	Y	Y	Y	N	Y	Y	Include
Li et al. [42]	Y	Y	Y	Y	Y	Y	Y	Y	Y	Y	Y	Include
Matthews et al. [43]	Y	Y	Y	Y	Y	Y	Y	Y	N	Y	Y	Include
Ng et al. [44]	Y	Y	Y	Y	Y	Y	Y	Y	N	Y	Y	Include
5	Y	Y	Y	Y	Y	NC	NA	Y	N	Y	Y	Exclude
Nissen et al. [45]	Y	Y	Y	Y	Y	NC	NA	Y	Y	Y	Y	Include
Ochoa et al. [46]	Y	Y	Y	Y	Y	Y	Y	Y	N	Y	Y	Include
6	Y	Y	Y	Y	Y	NC	NA	Y	NC	Y	Y	Exclude
Petricone et al. [47]	Y	Y	Y	Y	Y	Y	Y	Y	NC	Y	Y	Include
Sak‐Dankosky et al. [48]	Y	Y	Y	Y	Y	Y	Y	Y	N	Y	Y	Include
7	Y	Y	Y	Y	Y	Y	NC	NC	N	Y	Y	Exclude
Streck et al. [7]	Y	Y	Y	Y	Y	Y	Y	Y	N	Y	Y	Include
8	Y	Y	Y	Y	Y	Y	NC	NA	NC	Y	Y	Exclude
Tragantzopoulou et al. [49]	Y	Y	Y	Y	Y	Y	Y	Y	N	Y	Y	Include
Valente et al. [50]	Y	Y	Y	Y	Y	NC	Y	Y	NC	Y	Y	Include
9	Y	Y	Y	Y	Y	Y	NC	NC	N	Y	Y	Exclude
Wang et al. [51]	Y	Y	Y	Y	Y	Y	Y	Y	NC	Y	Y	Include
Zeng et al. [52]	Y	Y	Y	Y	Y	Y	Y	Y	N	Y	Y	Include
Zhou et al. [53]	Y	Y	Y	Y	Y	Y	Y	Y	N	Y	Y	Include

*JBI Critical Appraisal Checklist	
1	Is the review question clearly and explicitly stated?
2	Were the inclusion criteria appropriate for the review question?
3	Was the search strategy appropriate?
4	Were the sources and resources used to search for studies adequate?
5	Were the criteria for appraising studies appropriate?
6	Was critical appraisal conducted by two or more reviewers independently?
7	Were there methods to minimize errors in data extraction?
8	Were the methods used to combine studies appropriate?
9	Was the likelihood of publication bias assessed?
10	Were recommendations for policy and/or practice supported by the reported data?
11	Were the specific directives for new research appropriate?

**TABLE 2:** Main characteristics of the included studies.

First author (year)	Country of study	Included studies (n)	Review type	Research question	Cancer type	Main findings
Akter et al. (2023) [25]	Turkey (*n* = 6); Brazil (*n* = 1); South Africa (*n* = 1); USA (*n* = 3); Korea (*n* = 1); Iran (*n* = 1); Italy (*n* = 1); France (*n* = 1)	15	Integrative review Qualitative study using interpretive thematic synthesis	Factors influencing caregiver burden and QoL among cancer patient caregivers, identifying targets for tailored interventions.	Non‐specific	Higher perceived social support is associated with better QoL. Caregivers' QoL depends on caregiving burden, cohabitation, caregiver–patient relationship, and family roles.
Alfaro‐Diaz et al. (2022) [26]	USA (*n* = 8); UK (*n* = 1); Australia (*n* = 1); Iran (*n* = 2); Turkey (*n* = 2); France (*n* = 1); China (*n* = 1); India (*n* = 1); Norway (*n* = 1); Denmark (*n* = 1)	19	Quantitative systematic review	Characteristics and effectiveness of nursing interventions for adult cancer patients and families.	Non‐specific	Interventions focusing on the couple or family relationship produce more significant and lasting positive outcomes.
Applebaum et al. (2013) [27]	N/D	49	Quantitative systematic review	Psychosocial interventions for informal caregivers of cancer patients, including effectiveness and feasibility.	Non‐specific	Couple‐ or family‐focused interventions improve caregiver skills and produce more lasting positive effects. Psychoeducational and couple interventions enhance care and health, and reduce depression and anxiety.
Boele et al. (2019) [28]	US (*n* = 4); Australia (*n* = 1); Netherlands (*n* = 2); Iran (*n* = 1)	8	Qualitative synthesis of evidence from randomized controlled trials (RCTs) and quasi‐RCTs.	Effects of supportive interventions on caregiver well‐being, patient health, and cost‐effectiveness in brain or spinal cord cancer care.	Central nervous system (CNS) tumors	Reviewed interventions include various types (e.g., cognitive behavioral therapy, psychoeducation, coping skills training, self‐management, social network interventions) and delivery modes (face‐to‐face, web‐based). Some show beneficial effects in reducing psychological distress, enhancing caregivers' sense of mastery, and improving overall quality of life.
Brunt et al. (2023) [29]	USA (*n* = 5); Sweden (*n* = 1); Denmark (*n* = 1); Turkey; (*n* = 1); Canada (*n* = 1); France (*n* = 1)	10	Integrative review	Needs of informal caregivers of hematological malignancy patients post‐discharge.	Acute leukemia; multiple myeloma; Non‐Hodgkin lymphoma; Chronic myeloid leukemia; Acute lymphoblastic leukemia	Cancer affects the whole family, disrupting roles and intimacy, especially in couples and families with children. Caregivers are mostly female spouses.
Cai et al. (2021) [30]	Australia (*n* = 1); Singapore (*n* = 1); Ghana (*n* = 1); Canada (*n* = 1); USA (*n* = 1); N/d (*n* = 1)	6	Qualitative study using thematic synthesis	Impact of cancer caregiving on informal caregivers' QoL and strategies supporting their role.	Non‐specific	Caregivers adapt roles, prioritize patient needs, rely on social support to prevent isolation, and seek professional, informational, and emotional support.
Chen et al. [31]	UK (*n* = 2); USA (*n* = 3); Germany (*n* = 3); Sweden (*n* = 2); Australia (*n* = 3); Danimarca; (*n* = 1); Belgium (*n* = 1); Turkey (*n* = 1)	16	Qualitative meta‐synthesis	Experiences and needs of informal glioma caregivers throughout the disease trajectory.	Glioma	Caregivers of glioma patients need evolving informational support, rely on family, social, and professional resources, but may hesitate to share concerns.
Cochrane et al. (2021) [2]	USA (*n* = 21); Taiwan (*n* = 2); Hong Kong (*n* = 1); South Korea (*n* = 1); Brazil (*n* = 1); Netherlands (*n* = 1)	27	Quantitative systematic review	Factors associated with lung cancer caregiver distress.	Lung cancer	A dyadic approach reveals interdependent processes; caregiver–patient bonds and social support help mitigate the risk of depression and anxiety.
Coyne et al. (2020) [32]	USA (*n* = 45); Europe (*n* = 20)	73	Systematic quantitative literature review (Pickering method)	Family‐focused approaches addressing the needs of families during cancer treatment.	Non‐specific	Family communication supports patient and family health; communication‐focused interventions improve coping and overall QoL.
Frambes et al. (2018) [33]	USA (*n* = 9); Australia (*n* = 2); Canada (*n* = 1); Germany (*n* = 1); Netherlands (*n* = 1)	14	Quantitative literature review from RCTs only	Roles, needs, and health impacts of cancer caregivers based on supportive interventions and caregiving activities.	Non‐specific	Caregiving impacts lifestyle, work, and relationships; psychoeducational and CBT interventions enhance skills, communication, coping, and role mastery.
Gray et al. (2019) [34]	N/D	18	Narrative review	Prevalence, impact, and management of loneliness among cancer caregivers, including strategies to reduce it.	Non‐specific	Caregivers often lose contact with friends and social groups due to patient restrictions, leading to isolation; interventions improve social skills, provide support, increase social interactions, and address maladaptive social cognition, with CBT and group therapies particularly effective.
Hasdenteufel et al. (2022) [35]	Japan (*n* = 4); Canada (*n* = 3); Australia (*n* = 3); Germany (*n* = 1); Israel (*n* = 1); Italy (*n* = 2); Norway (*n* = 1); USA (*n* = 1); Taiwan (*n* = 2)	18	Systematic literature review (thematic synthesis)	Biopsychosocial and existential factors influencing grief in caregivers during the palliative phase.	Non‐specific	Perceived social support and professional discussions aid grief adaptation, while ambivalent or conflictual patient relationships predict poor adjustment.
Heckel et al. (2019) [36]	USA (*n* = 19); Australia (*n* = 14); UK (*n* = 7); Netherlands (*n* = 2); Canada (*n* = 1); Nigeria (*n* = 1); Serbia (*n* = 1)	45	Narrative synthesis review	Characteristics, satisfaction, and effectiveness of cancer helplines for caregivers.	Non‐specific	Frequent callers to oncology helplines are distressed middle‐aged or older, educated, married Caucasian women who initially seek information and later emotional support.
Hoeck et al. (2015) [37]	Denmark (*n* = 1); USA (*n* = 2); Sweden (*n* = 1); Norway (*n* = 1); Australia (*n* = 1); UK (*n* = 7); Canada (*n* = 2); Turkey (*n* = 1)	16	Narrative review	Provision of psychosocial support to caregivers, considering individual needs, involvement of significant others, and providers.	Lung cancer; Gynecological cancer	Caregivers need information, reassurance, assessment, connection, emotional support, and side‐effect management; feeling heard is essential across cancer types.
Jones et al. (2022) [38]	USA (*n* = 10); Australia (*n* = 4); Denmark (*n* = 2); Italy (*n* = 2); Canada (*n* = 1); Netherlands (*n* = 1); UK (*n* = 1)	21	Systematic literature review (narrative synthesis)	Carer‐reported benefits of supportive care strategies for adults with high‐grade glioma.	Glioma	Caregivers value peer support and emotional care from healthcare professionals, which provide a safe space, validate their role, and reduce isolation.
Kim et al. (2023) [39]	Netherlands (*n* = 9); USA (*n* = 3); Portugal (*n* = 2); Israel (*n* = 1); China (*n* = 1); UK (*n* = 1); Australia (*n* = 1)	18	Systematic review and meta‐analysis	Dyadic processes affecting psychological health and health‐related QoL (HRQL) in colorectal cancer (CRC) patients and caregivers.	Colon cancer	Psychological health in dyads is interdependent; caregiver social support affects distress, highlighting the need for dyadic interventions.
Kleine et al. (2019) [40]	USA (*n* = 5); Canada (*n* = 3); Norway (*n* = 1)	9	Systematic literature review (thematic synthesis)	Types and effects of psychological interventions for intimate partners of cancer patients.	Non‐specific	Psychological interventions for partners of cancer patients demonstrate some positive effects for both partners and patients; however, the evidence is limited by moderate‐to‐weak methodological quality and small sample sizes.
LeSeure et al. (2015) [41]	N/D	18	Systematic review and meta‐synthesis	Experiences and responses of caregivers to the emotional, practical, and social demands of caring for cancer patients.	Non‐specific	Caregivers experience life changes post‐diagnosis; relationship quality sometimes improves. Key challenges include cancer communication, accepting help, normalizing care, seeking balance, and relying on family support.
Li et al. (2014) [42]	USA (*n* = 25); Australia (*n* = 1); Canada (*n* = 1); Netherlands (*n* = 1); Israel (*n* = 1); South Korea (*n* = 1); Taiwan (*n* = 1)	31	Literature review (Inductive Content analysis)	Role of mutuality in shaping relationships and adjustment of cancer patients and spousal caregivers.	Non‐specific	Better couple communication and constructive discussions correlate with reduced distress and improved marital adjustment; frequent talks lower anxiety, though some partner traits hinder communication.
Matthews et al. (2023) [43]	Australia (*n* = 6); USA (*n* = 4) N/d (*n* = 5)	15	Systematic review (narrative synthesis)	Psychosocial experiences of informal caregivers of oropharyngeal cancer patients post‐treatment.	Head and neck cancer	Most caregivers are spouses or partners, facing relational changes and unmet needs in information, communication, and psychological support; peer support and shared caregiving roles are vital amid dyadic tensions and limited social networks.
Ng et al. (2023) [44]	USA (*n* = 9); Netherlands (*n* = 3); Israel (*n* = 3); Australia (*n* = 2); South Korea (*n* = 1); Sweden (*n* = 1); Switzerland (*n* = 1)	20	Systematic review (narrative synthesis)	Gender differences affecting the impact of cancer caregiving on spouses.	Non‐specific	Female spousal caregivers face greater personal and social restrictions and lower marital satisfaction, and are more reluctant to seek support, while male caregivers report higher personal expenses and social impacts.
Nissen et al. (2016) [45]	USA (*n* = 4); Canada (*n* = 4); Italy (*n* = 2); Australia (*n* = 1); France (*n* = 1); Turkey (*n* = 1)	13	Systematic review and meta‐analysis	Influence of anxious/avoidant attachment on psychosocial variables in cancer patients and caregivers.	Non‐specific	Higher insecure attachment levels moderately link to lower social support; insecure caregivers struggle to seek attention and care from their environment.
Ochoa et al. (2020) [46]	USA (*n* = 60)	60	Systematic literature review (narrative synthesis)	Current state of research on caregiving QoL to guide interventions.	Non‐specific	Perceived social support correlates with quality of life; lack of support worsens caregiver burden and health. Younger caregivers adapt worse, spousal caregivers report poorer mental health, and female caregivers prioritize support for anxiety and stress more than males.
Petricone‐Westwood et al. (2016) [47]	UK (*n* = 1); Sudan (*n* = 1); Australia (*n* = 3); Canada (*n* = 5); USA (*n* = 8); Italia (*n* = 1)	19	Systematic literature review (thematic synthesis)	Experiences and well‐being of caregivers of ovarian cancer patients.	Ovarian cancer	Social support is a major unmet need for caregivers, who find comfort in peer letters. Caregiving reorganizes family dynamics, with caregiver well‐being linked to patient psychological health and mixed marital relationship changes.
Sak‐Dankosky et al. (2022) [48]	USA (*n* = 18); Australia (*n* = 4); Denmark (*n* = 2); Iran (*n* = 1); Canada (*n* = 4); Singapore (*n* = 2); China (*n* = 1); UK (*n* = 2); Nigeria (*n* = 1); Taiwan (*n* = 1); Netherlands (*n* = 1)	37	Systematic literature review (narrative synthesis)	Psychosocial interventions improving caregiver well‐being, including methodological and clinical characteristics.	Non‐specific	Various interventions—online, phone, or face‐to‐face—successfully improve caregiver health; addressing specific informational needs and including social support and psychoeducation are key for clinical effectiveness.
Streck et al. (2020) [7]	USA (*n* = 4); Turkey (*n* = 1); Egypt (*n* = 1); Israel (*n* = 1); Netherlands (*n* = 1); Denmark (*n* = 2)	10	Systematic literature review (narrative synthesis)	Dyadic experience of depression among couples with one partner having breast cancer.	Breast cancer	Support from friends, family, and spouses protects breast cancer patients' partners from depression; low perceived social support correlates with higher depression, with reciprocal influences between patients and partners.
Tragantzopoulou et al. (2024) [49]	USA (*n* = 7); UK (*n* = 2); Australia (*n* = 2); Canada (*n* = 1); Germany (*n* = 1); Indonesia (*n* = 1); Netherlands (*n* = 1); China (*n* = 1); Ireland (*n* = 1)	17	Qualitative meta‐synthesis	Experiences and needs of caregivers of lung cancer patients.	Lung cancer	Many caregivers struggle with multiple roles, emotional challenges, and uncertainty; support groups may increase stress, and online resources provide information but cannot replace personalized professional guidance and reassurance.
Valente et al. (2021) [50]	USA (*n* = 11); France (*n* = 2); Canada (*n* = 1); Australia (*n* = 1); South Korea (*n* = 1); Turkey (*n* = 1)	17	Systematic review	Relationship dynamics and dyadic processes in couples during breast cancer treatment.	Breast cancer	Illness affects relational functioning across dimensions; constructive communication, dyadic coping, and informal social networks predict positive cancer adaptation, enhancing self‐esteem and reducing couple stress, especially in breast cancer.
Wang et al. (2018) [51]	USA (*n* = 9); China (*n* = 7); Australia (*n* = 5); Netherlands (*n* = 5); Canada (*n* = 4); Japan (*n* = 3); Taiwan (*n* = 3); UK (*n* = 2); Denmark (*n* = 2); Hong Kong (*n* = 2); Italy (*n* = 1); France (*n* = 1); South Korea (*n* = 1); Spain (*n* = 1); Indonesia (*n* = 1); India (*n* = 1); Bangladesh (*n* = 1); Czech republic (*n* = 1)	50	Systematic review (Content and Descriptive analysis)	Unmet care needs and associated variables in advanced cancer patients and informal caregivers.	Head and neck cancer	Social support needs peak post‐treatment, especially after surgery; treatment‐related dysphagia and taste loss hinder patient and caregiver social activities, highlighting the need for comprehensive caregiver support, including psychological aid and practical skills training.
Zeng et al. (2022) [52]	Ireland (*n* = 4); Australia (*n* = 3); UK (*n* = 2); USA (*n* = 2); Sweden (*n* = 1); Italy (*n* = 1); New Zealand (*n* = 1); China (*n* = 1); Thailand (*n* = 1); Canada (*n* = 1); India (*n* = 1); Indonesia (*n* = 1)	20	Systematic review and meta‐synthesis	Experiences of family caregivers providing care to head and neck cancer patients.	Head and neck cancer	Dyadic communication difficulties and patient psychological health affect social interactions; some caregivers find positive meaning in their role and value peer support, but often suppress their own needs to protect others, limiting received support.
Zhou et al. (2023) [53]	USA (*n* = 9); Canada (*n* = 3); Australia (*n* = 1); China (*n* = 1)	14	Systematic review	Characteristics and outcomes of couple‐based communication interventions in cancer care.	Non‐specific	Addressing symptom management, treatment, caregiving, prognosis, role changes, marital relations, and social/family issues is crucial for couples' psychosocial adaptation to cancer; communication‐focused interventions enhance this adaptation.
Zhu (2022)	USA (*n* = 8); Canada (*n* = 3); Germany (*n* = 3); Taiwan (*n* = 2); Hong Kong (*n* = 1); Singapore (*n* = 1); UK (*n* = 2); Italy (*n* = 2); Netherlands (*n* = 1); Ghana (*n* = 1); Indonesia (*n* = 1); Australia (*n* = 1)	26	Systematic review and meta‐synthesis	Experiences of family caregivers providing care to patients with advanced cancer.	Non‐specific	Caregivers often experience social withdrawal and isolation, relying on family, friends, and colleagues for spiritual, financial, and practical support, while commonly concealing emotions; a frequent positive change is strengthened emotional bonds with the patient.

### 3.2 Narrative Synthesis of the Included Studies

In line with our main objective, we conducted a high‐level narrative synthesis on the included studies regarding the role of social support in the experience of informal cancer caregivers. We identified three most recurrent thematic areas: (i) the role of social support in shaping caregivers' outcomes, including their psychological well‐being, adaptation to the caregiving role and difficulties in receiving and seeking help (3.2.1); (ii) changes in interpersonal relationships, including the relationship with the patient (3.2.2); and (iii) support needs and psychosocial interventions designed to address them (3.2.3).

#### 3.2.1 The Effects of Social Support on the Psychological Well‐Being

The included studies are consistent in emphasizing the importance for the caregiver of having someone available who can talk and listen [29, 37, 38]. Support primarily comes from family, friends, neighbors, and colleagues, who are considered essential sources [30, 41, 54]. These interactions help caregivers find ways to cope with stress and negative emotions [52]. e.g., they might find emotional support by sharing the burden, listening, and experiencing the presence of the other person or they can receive practical help with domestic chores, errands, or alternating care responsibilities [41, 47, 54]. Independently of the type of help, perceived social support is linked to an overall decreased of caregiving burden [29, 41, 51]. Overall, adequate support helps caregivers maintain continuity in their own lives while managing caregiving demands [38, 47], thereby alleviating the caregiving load and reducing depressive symptoms [2]. Conversely, inadequate support is associated with poorer quality of life, including increased fatigue, sleep disturbances, and spousal distress [25, 39, 46, 47], potentially compromising the caregiver's overall health [46]. A lack of help or perceived neglect can lead caregivers to feel sad, discouraged, and isolated, often resulting in them managing care alone [30]. This isolation contributes to loneliness, depression, and an increased caregiver burden [30, 34, 46]. This is especially true in the pre‐bereavement phase, where a lack of social support can lead to poor adjustment and complicated grief [35]. Difficulties discussing cancer in social contexts can also exacerbate feelings of isolation, particularly during the patient's last year of life [34]. Caregivers reporting more relational problems with the patient often perceive less support and experience higher levels of depression [7]. Finally, our synthesis confirms the relevance of gender differences: female caregivers place greater value on support for managing anxiety and stress. At the same time, female spouses tend to report less social support, higher stress levels and a stronger sense of over‐responsibility than male caregivers [31, 46]. Gender disparities also appear in daily life changes, including social and work habits: women are more likely than men to change jobs, reduce hours, or retire early, and tend to delegate care tasks less [17, 18].

##### 3.2.1.1 Social Support Promotes Adaptation to the Caregiver Role

Adapting to the demanding role of an informal caregiver after a cancer diagnosis presents significant personal challenges, often marked by self‐neglect, declines in overall quality of life, shifts in personal identity, and broader changes within the family system [25, 29, 30, 41, 46, 54]. The narrative synthesis of the included studies supports the view that social support from both informal and formal networks is essential for promoting successful adaptation to these challenges [31, 41, 43, 46, 50, 52]. Specifically, perceived social support facilitates personal growth in the caregiving role, helps maintain hope, and supports relationship quality during treatment and survivorship [31, 35, 46, 47].

##### 3.2.1.2 Difficulties in Accepting and Seeking Support

Although social support is widely recognized as positively influencing the caregiving experience, current evidence shows that caregivers frequently face significant barriers to accepting or actively seeking help. These obstacles can arise from internal factors, including a sense of sole responsibility, reluctance to seek help, fear of being misunderstood, or efforts to hide distress [31, 41, 44, 52], as well as relational patterns, such as insecure attachment that hinder effective help‐seeking [2, 45, 46]. External and situational challenges also play a role, including practical issues like lack of time, negative perceptions of formal support services [49], fear‐driven social withdrawal [34], and communication difficulties within the family or social network [30, 31]. Consequently, caregivers often delay seeking assistance until they are already in significant distress [36].

#### 3.2.2 Changes in Interpersonal Relationships

Current evidence consistently shows that caregiving substantially alters caregivers' social relationships, with women often experiencing a greater change [44]. Caregivers are often required to reduce work hours or forgo careers and leisure activities [29, 31, 33, 34]. This is directly related to the significant time commitment required, especially for home‐based care or when living with the patient, which reduces opportunities for social engagement [25, 34]. This frequently leads to tangible changes in social networks, including loss of contact with friends and previous social habits, often contributing to the feelings of loneliness [34]. Additionally, external factors, such as clinical routines, patient symptoms and functional decline can further restrict social participation and caregiver freedom [31, 34, 51]. Despite these widespread challenges, some caregivers also report positive relational changes, such as strengthened emotional bonds with partners or enhanced family teamwork and unity [54].

##### 3.2.2.1 The Relationship With the Patient

The relationship between caregiver and patient undergoes significant changes and role renegotiation, a finding consistently reported across reviews [37, 39, 41, 42, 43, 44, 47, 50, 51, 52, 54]. Caregivers might feel they have assumed the role involuntarily, with difficulties often arising from changes in relationship dynamics compared to the pre‐diagnosis period, sometimes shifting toward a parental role [43]. Within these relationship dynamics, strong interdependencies have been reported within caregiver‐patient dyads regarding well‐being and adaptation [7, 37, 39, 41, 42, 43, 44, 46, 47, 48, 50, 51, 52, 53, 54], with reciprocal influences observed in areas such as depression [7, 39, 42, 46, 53]. Renegotiating established roles can cause tension [29] and caregivers may perceive a loss of equality with the patient [31]. This can be particularly evident when living together with the patient: several reviews acknowledge that in some cases informal caregivers co‐reside with the patient [25, 29, 34, 54]; however, the included studies do not directly address the specific experience of living together with the patient, expect for spousal caregivers. In this case, marital satisfaction may decline [44], often accompanied by poor communication, role imbalance, and loss of intimacy as common stressors [29, 31, 43, 44] and a higher risk of depression compared to caregivers in other types of relationships [2, 25, 43, 46, 47]. On the contrary, constructive communication, mutual support, and dyadic coping strategies are linked to better psychological outcomes [2, 32, 42, 50, 53]. However, despite these hardships, the caregiving experience can also foster positive relational changes. Some caregivers report strengthened bonds, greater family closeness and teamwork, personal or marital growth, a sense of fulfillment from providing care, and a deeper appreciation of shared time [41, 52, 54].

#### 3.2.3 Support Needs and Psychosocial Interventions

Caregivers require specific support to deal with the significant emotional distress they face [49]. As part of this support, they value emotional validation and informal communication from healthcare professionals [38]. Current evidence consistently shows that caregivers have a strong initial need for comprehensive information across multiple domains (e.g., illness, symptom management, and care expectations), often prioritizing this need over emotional support when first seeking help [27, 30, 36, 51]. Therefore, effective interaction with healthcare professionals is critical [54]. Conversely, inadequate or insufficient communication is a major source of stress and a barrier to meeting their needs [54], highlighting the importance of proper communication skills when engaging with caregivers.

##### 3.2.3.1 Psychoeducational Interventions

Interventions can initially provide guidance on the illness, symptom management, and care expectations. Despite the importance on offering such information, interventions focused solely on this type of support showed limited impact [27, 48]. However, when these interventions are combined with psychoeducation, such as training in stress management and coping strategies, they generally lead to positive outcomes, including significant improvements in caregivers' quality of life [27, 28, 33, 48]. These programs can normalize caregivers “experiences, strengthen their confidence, improve quality of life, and enhance self‐efficacy in providing care, although some benefits may diminish after 6 months [26, 28, 33]. For example, interventions targeting caregiving competence and problem‐solving skills have been shown to significantly reduce care burden, providing support in managing new daily care tasks [27]. Additionally, interventions targeting social and interpersonal skills have effectively reduced loneliness and isolation, particularly among older caregivers [34]. Structured and time‐limited interventions have been shown to be both feasible and beneficial [27, 38], also when delivered through various modalities including telemedicine [27, 36, 38] and telephone helplines, which help bridge communication gaps with healthcare professionals [27, 36]. The positive outcome of the intervention seems to be independent of whether the delivery is online, by phone, or face‐to‐face [48]. Ultimately, effective support requires repeated needs assessments and should be continuous, personalized, and co‐planned with the caregiver [37, 38]. In sum, current research suggests that combining psychoeducation, skills development, and counseling may be an effective, evidence‐based approach that leads to more sustained improvements in caregivers” quality of life [33, 34, 39].

##### 3.2.3.2 Dyadic Interventions: Communication as a Coping Strategy

In addition to interventions that directly target caregivers, those that focus on communication between caregivers and patients (especially couples) may also play a key role in reducing distress and improving relationship quality [2, 28, 32, 42, 47, 50, 53], given the interdependencies between the two. e.g., while poor communication has been identified as a risk factor for complicated grief, interventions supporting communication can facilitate bereavement adjustment [35]. Open communication fosters mutual adaptation, lowers distress, enhances relationship satisfaction, and helps navigate tensions from role renegotiation [2, 29, 31, 32, 42, 47, 50]. Overall, current research shows that dyadic interventions focusing on communication and mutual support provide a valuable support for caregivers [26, 39, 40, 53]. Such interventions have been shown to be more effective than individual approaches for dyadic functioning [27, 33], facilitating help‐seeking behaviors and improving well‐being within the family system [32].

## 4 Discussion

This umbrella review aimed to provide a high‐level narrative synthesis of evidence from systematic reviews and meta‐analyses on the role of social support in shaping the experience of informal caregivers of cancer patients. In response to the growing prevalence of cancer and the expected increase in caregiver burden [1, 3, 5], the review sought to clarify how social support influences caregiver outcomes, to identify areas of consensus and disagreement, and to offer guidance for future research, practice and policy.

Overall, the findings consistently confirm social support as a central factor of caregivers’ psychological well‐being, adaptation to the caregiving role, and quality of life [2, 29, 31, 41, 51]. Across the included reviews, spanning diverse caregiving relationships, methodological approaches and cancer types (e.g., breast, lung, colorectal, glioma, and head and neck cancers), higher levels of perceived social support were robustly associated with lower caregiver burden, reduced depressive symptoms, and better overall adjustment [2, 29, 41]. Informal sources of support (e.g., family members, friends, and peers) emerged as essential in helping caregivers cope with emotional distress and practical demands [30, 37, 52, 54]. Specifically, instrumental and emotional support have been shown to reduce the likelihood or severity of caregiver burden and enhancing overall well‐being [29, 49]. This convergence across reviews including primary studies conducted in different countries and care contexts further reinforces social support as a protective factor in informal oncology caregiving. Our findings support the view that social support promotes well‐being by offering practical assistance with daily tasks and by providing stable psychological resources, such as a sense of security and self‐esteem, as well as by buffering the negative effects of stressors [55]. This stress‐buffering mechanism operates by shaping cognitive appraisals, reducing perceived threat, and facilitating effective coping and problem‐solving, in line with transactional models of stress [28].

Despite its importance, caregivers frequently experience difficulties in accepting or actively seeking support, a phenomenon frequently reported in the current literature [31, 41, 44, 52]. Reviews consistently show internal barriers such as a strong sense of responsibility, reluctance to burden others, emotional suppression, or insecure attachment patterns [2, 31, 41, 45, 46, 52], alongside external barriers including time constraints, limited access to services, and negative perceptions of formal support [30, 34, 49, 56]. The difficulties in accepting or seeking help can be understood through the concept of distribution of dependence applied to the caregiving experience [8, 57]. Depending more on the support of others significantly impacts the caregiver's experience, potentially increasing anxiety and depression, especially if the caregiver previously relied heavily on the patient to satisfy their needs. Investigating the distribution of dependence of the caregiver can help better understand their adaptation to the caregiving role. Factors such as a self‐sufficiency or a primary reliance on oneself can directly influence whether external help is seeked. Caregivers may believe they should cope independently or feel guilty within the caregiver–patient relationship, fearing a sense of betrayal when prioritizing their own needs. Caregivers may avoid seeking help to preserve their self‐sufficient role. Help‐seeking avoidance by caregivers could also be understood through the concept of relationship ambivalence, where individuals simultaneously experience support and stress from the same close relations, such as intimate friends or the patient [58]. This is particularly applicable to the caregiver‐patient relationship, as the patient's own loss of autonomy and heightened vulnerability may transform them into a source of stress while limiting their ability to provide support.

The results further show strong consensus on the disruptive impact of caregiving on interpersonal relationships [25, 29, 31, 33, 34]. Caregivers often reduce work participation, withdraw from leisure activities, and experience shrinking social networks, contributing to loneliness and social isolation [25, 34]. These effects appear particularly pronounced among women, especially spousal caregivers, showing gender differences highlighted in several included reviews [31, 44, 46]. However, the evidence also consistently acknowledges that caregiving can generate positive relational changes, including strengthened emotional bonds, enhanced family cohesion, and shared meaning‐making [41, 52, 54].

In line with these results, a common theme identified in various studies is the renegotiation of caregiver and patient roles. Reviews consistently report strong interdependencies between patient and caregiver well‐being [7, 37, 39, 42, 44, 47, 48, 50, 51, 52, 53, 54]. Importantly, spousal caregivers are reported to be more vulnerable than other caregiver types, experiencing greater impacts on quality of life and mental health. The specific challenges faced by spousal caregiver–patient dyads have been conceptualized within the Adult Attachment Theory [2, 45, 46, 59]. This theory posits partners as a “secure base” that fosters personal exploration and growth. Within this context, supportive behaviors include validating concerns, expressing confidence in one's partner and offering emotional and instrumental assistance, highlighting the importance of effective interactions and interpretation of help‐seeking behaviors within caregiver–patient relationships. Overall, the consistency of these findings supports the conceptualization of caregiving not only as an individual experience but as a fundamentally relational one.

With respect to support needs and interventions, the evidence shows broad agreement that caregivers initially prioritize informational support [27, 30, 36, 51]. However, reviews converge in suggesting that interventions focused solely on offering information are often insufficient [27, 48]. Instead, interventions combining psychoeducation, skills training, emotional support, and counseling are more consistently associated with improvements in caregiver quality of life, self‐efficacy, and burden reduction [26, 27, 28, 33, 48], independently of the delivery formats (e.g., face‐to‐face, telephone, online) [27, 36, 38, 48]. Dyadic and family‐based interventions, particularly those centered on communication, also show more sustained effects than individual approaches [26, 28, 32, 40]. This result is in line with a dyadic illness management perspective, which views illness as a shared phenomenon, emphasizing transactional, interdependent dynamics where each member's health co‐varies [60]. According to this perspective, sharing emotions and fostering mutuality in the relationship allows both caregiver and patient to maintain a sense of self within the relationship despite drastic role changes.

### 4.1 Limitations

Despite these areas of strong consensus, some inconsistencies and limitations remain. The included studies exhibit a high degree of heterogeneity, a limitation inherent to the umbrella review methodology. This heterogeneity comes from the inclusion of diverse populations that differ, for example, in cancer type, geographic or cultural context. For example, most of the reviews included primary studies conducted in Western countries, especially United States and European countries. By contrast, countries in Asia, Africa, and the Middle East are represented to a much lesser extent. These findings indicate that most studies are embedded within Western cultural contexts. This imbalance should be considered when interpreting the results, as cultural norms, healthcare systems, family structures, and caregiving roles may differ across countries, potentially limiting the generalizability of the findings to non‐Western populations. In addition, the specific experience of living together with the patient was not directly examined, as none of the reviews included primary studies focusing exclusively on caregivers co‐residing with the patient. Finally, none of the included studies directly addressed the specific caregiving experience of LGBT + people, which may face distinct challenges related to minority stress, stigma, and disclosure in healthcare settings, which can affect access to support, communication with professionals, and psychological well‐being.

### 4.2 Implications for Research, Practice, and Policy

The findings of this umbrella review suggest several directions for future research. There is a need to move beyond establishing the importance of social support toward identifying which forms of support are most effective at different stages of the cancer trajectory and for different caregiver groups. Longitudinal designs can be helpful to assess the sustainability of intervention effects and to capture evolving support needs over time during the illness trajectory. Future reviews and primary studies should also more systematically examine gender, relationship type, sexual orientation, gender identity and cultural context as factors shaping the role of social support in the caregiving experience [17, 20, 31, 39, 46].

From a practice and policy perspective, the consistent evidence highlights the importance of recognizing informal caregivers as key figure within oncology care [1, 3, 5]. Therefore, healthcare systems should implement routine assessment of caregiver needs and invest in caregiver‐centered communication training for professionals [27, 33, 38, 48]. The analysis of the results of previous studies [16, 32, 33] suggest that policies that promote early intervention, facilitate help‐seeking, and support dyadic and family‐based approaches may help reduce caregiver burden and improve outcomes for both caregivers and patients.

## Author Contributions

S.C. conceived the study, supervised the project and collaborated with M.O. in the selection and review of the included studies. M.O. conducted the literature search, data extraction, and analysis. A.G. contributed to the writing and critical revision of the manuscript. All authors contributed to the writing of the manuscript, read and approved the final manuscript.

## Conflicts of Interest

The authors declare no conflicts of interest.

## Data Availability

The authors have nothing to report.
